# Responsive Hydrogel‐Based Modular Microrobots for Multi‐Functional Micromanipulation

**DOI:** 10.1002/smll.202404311

**Published:** 2024-07-23

**Authors:** Liyuan Tan, David J. Cappelleri

**Affiliations:** ^1^ School of Mechanical Engineering Purdue University West Lafayette IN 47907 USA; ^2^ Weldon School of Biomedical Engineering Purdue University West Lafayette IN 47907 USA

**Keywords:** microrobots, modular, hydrogel

## Abstract

Microrobots show great potential in biomedical applications such as drug delivery and cell manipulations. However, current microrobots are mostly fabricated as a single entity and type and the tasks they can perform are limited. In this paper, modular microrobots, with an overall size of 120 µm × 200 µm, are proposed with responsive mating components, made from stimuli‐responsive hydrogels, and application specific end‐effectors for microassembly tasks. The modular microrobots are fabricated based on photolithography and two‐photon polymerization together or separately. Two types of modular microrobots are created based on the location of the responsive mating component. The first type of modular microrobot has a mating component that can shrink upon stimulation, while the second type has a double bilayer structure that can realize an open and close motion. The exchange of end‐effectors with an identical actuation base is demonstrated for both types of microrobots. Finally, different manipulation tasks are performed with different types of end‐effectors.

## Graphical abstract

Magnetic modular microrobots that can change their end‐effectors to achieve different functions are presented. The two modules, the magnetic base and the end‐effector, are connected by a responsive mating component made from hydrogel materials, fabricated with two‐photon polymerization. The modular microrobots successfully demonstrate with their ability to exchange end‐effectors and perform micromanipulation applications.

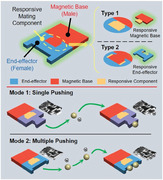

## 1 Introduction

Untethered microrobots have been found promising for biomedical applications, such as drug delivery,^[^ ^1^, ^2^, ^3^ ^]^ single cell/tissue manipulation,^[^ ^4^, ^5^ ^]^ and single‐cell studies.^[^ ^6^ ^]^ Other potential applications like micromanipulation of micro objects for low‐cost manufacturing are also popular.^[^ ^7^ ^]^ In the past decade, many solutions have emerged for micromanipulation tasks with microrobots. For example, helical microswimmers have been fabricated for cargo transport under a rotating magnetic field.^[^ ^8^ ^]^ Force‐sensing microrobots are achieved for safe manipulation with controlled force using a gradient magnetic field.^[^ ^9^, ^10^ ^]^

In the past few years, the rapid development of smart polymers, such as hydrogels and liquid crystalline polymers, have introduced new design scenarios for microrobots.^[^ ^11^, ^12^ ^]^ For example, microcrawlers,^[^ ^13^ ^]^ helical microswimmers,^[^ ^14^, ^15^ ^]^ and microgrippers^[^ ^16^, ^17^, ^18^ ^]^ have been fabricated with hydrogels. Liquid crystalline polymers have also been used to achieve microcrawlers with feet.^[^ ^19^ ^]^ These smart polymers are able to deform after fabrication under stimuli, such as temperature, light, and chemical changes, which provides an additional freedom of design in addition to geometry.^[^ ^11^, ^20^ ^]^ The material response allows for the introduction of advanced functionalities. Compared with microrobots obtained from traditional stiff materials, hydrogels are soft and responsive so that they can be used to achieve microswimmers with adaptive locomotion, either passive or active.^[^ ^21^, ^22^ ^]^

Fabrication of modular microrobots that have multiple micro‐components achieving different tasks is one of the targeted goals in this area.^[^ ^23^, ^24^ ^]^ Achieving modular microrobots from sub‐components could lead to more advanced and optimal microrobots.^[^ ^23^, ^25^ ^]^ However, it was said that the realization of these modular microrobots is restricted due to the lack of materials and techniques required to solve the sophisticated design of microrobots.^[^ ^26^, ^27^ ^]^ Therefore, even though integrated multi‐functional microrobots, with different modules for various functions, are desired, achieving such microrobots is still challenging and complex considering that multiple materials will be used to achieve different functions. The compatibility of different materials should also be considered during the fabrication and thus the difficulty will increase dramatically as more functions are required. Moreover, limited by the stiffness of the materials previously used for microrobot fabrications, modular microrobots that can maintain both their individual and assembled modes without continuous stimulation are hard to achieve. Because of the above reasons, current research is focused on modular microrobots that can perform assembly and disassembly of multiple modules/sub‐units to achieve multi‐functionalities. For example, modular microswimmers were achieved using the magnetic dipole–dipole interaction between microbead subunits.^[^ ^28^ ^]^ However, the assembled states vary from one another because of the inconsistent magnetic moment of the base module, as well as the subunits. Moreover, since both the base module and the subunits are actuated under a global field, the assembly process is challenging because their swimming states must be controlled in a relative manner. Similarly, another type of modular microswimmer was also developed with patterned subunits.^[^ ^29^ ^]^ Those subunits are assembled into the final microswimmers through the magnetic dipole‐dipole interactions. The assembled microswimmers can also aggregate into a larger one as a result of the interaction. However, the reversibility of the assembly has not been discussed. Recently, modular microrobots have been developed for complex assemblies achieving gear structures, snake‐shaped structures, and micro‐vehicles.^[^ ^30^ ^]^ The locomotion and the assembly process are achieved by lifting the parts with a bubble generated optothermally. However, the locomotion of the microrobots is realized through an inefficient approach as the bubble is generated discretely. Recently, microstructures with assembly capabilities were fabricated taking advantage of dielectrophoretic forces generated by specially designed structural features under an electric field.^[^ ^31^ ^]^ These non‐magnetic microstructures are able to move under magnetic fields when they are incorporated with magnetic spheres which are assembled into the microstructures via the dielectrophoretic forces. However, the modular microrobots existing are not versatile and their functions are limited after fabrication.

By taking advantage of the responsiveness of hydrogels, that deform upon stimulation, self‐assembly with multiple subunits has been achieved. For example, self‐assembly of specially designed circular/rectangular discs at the air/water interface is achieved in Ref. [32, 33] The attraction forces are induced by photothermal deformations of the hydrogel discs. The locomotion of the discs can be obtained by moving a light between two discs. However, these subunits can only work at the air/water interface and the assembled structures are not able to move except for the case with only two subunits and a moving light. A recent study also uses hydrogel to achieve a modular microrobot by making different functional components at the same time by an aniso‐electrodeposition method.^[^ ^27^ ^]^ This method provides a one‐step fabrication process for modular microrobots that can perform propulsion, grasping, and object delivery. However, these microrobots still cannot exchange modules and a new microrobot is required once the gripping component is dissolved after delivery.

Even though there is a growing number of investigations on hydrogel‐based microrobots, modular microrobots with orthogonal actuation over the locomotion and the assembly process under a gradient magnetic field have not been explored. The number of reports on modular microrobots is also limited. Some applications of assembly only achieved the assembly process itself, not achieving any advanced functionalities by using different modules of the modular microrobots. Therefore, inspired by the computer numerical control (CNC) machine that can change their cutters to perform various tasks for manufacturing, multi‐functional modular microrobots with exchangeable end‐effectors are developed as illustrated below.

In this paper, we fabricate modular microrobots using the method of photolithography and two‐photon polymerization (TPP) with a footprint of 120 µm × 200 µm. The modular microrobots contain two parts: an end‐effector and a magnetic base. The end‐effector and the base are connected by a responsive mating component (RMC), which is achieved by a commonly used TPP‐polymerizable hydrogel. The hydrogel material has been applied to microrobots such as microswimmers^[^ ^34^ ^]^ and microcrawlers^[^ ^35^, ^36^ ^]^ using bilayer structures to provide the microrobots with the capability of swimming or crawling. Differently, the hydrogel here is adopted for the RMC for connecting different modules while a gradient magnetic field pulls around the microrobots. Depending on the location of the RMC, the modular microrobot can be categorized into two types: the first has an RMC (single‐layer design) as part of the magnetic base while the second one has an RMC (bilayer design) with the end‐effector. The RMCs show a significant size shrinkage in water and have a size slightly larger than designed in an environment containing 40% of water and 60% of ethyl lactate (EL). Both types of modular microrobots are investigated and validated for the mating process. Moreover, the proposed modular microrobots are used to achieve different micromanipulation tasks. The proposed modular microrobot separates the difficulties that will be encountered in manufacturing integrated multi‐functional microrobots into the magnetic base and the end‐effector. While the magnetic base achieves the locomotion of the microrobot, the design of the end‐effector is provided with more freedom of design without considering the requirements for locomotion.

## 2 Results and Discussion

### 2.1 Design Overview

**Figure 1:**
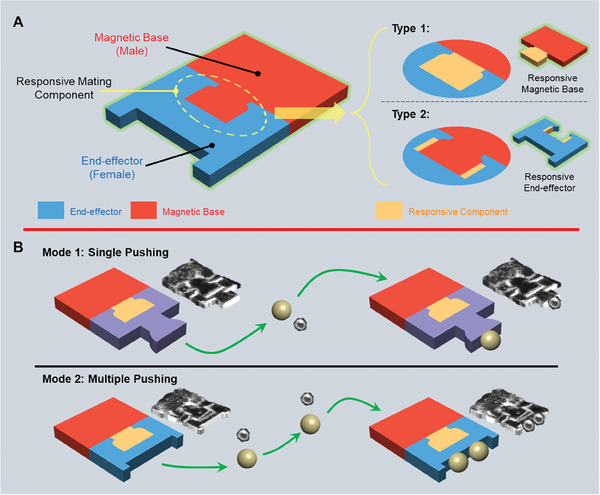
Schematic of the modular microrobots connected by an RMC. A) General design of modular microrobots with male and female features. The location of the RMC defines the microrobot types: Type 1 = base; Type 2 = end‐effector. B) Different modes of modular microrobots achieved with different types of end‐effectors.

The modular microrobots are designed with a male‐female connection between different modules as shown in **Figure** **1**A. In this paper, the male feature is placed with the magnetic base while the female feature is on the end‐effector. For the proposed modular microrobots, the attachment and detachment of different modules are achieved via the RMC where the male and female parts are connected as illustrated in the yellow circular region. Based on where the RMC is, the designs can be separated into two types. The RMC of the Type 1 microrobot is fabricated with the magnetic base, while the RMC for Type 2 is made with the end‐effectors. The RMC of the Type 1 microrobot is the male feature that is able to shrink upon stimulation and in order to detach from the end‐effectors. For Type 2, the RMS is part of the female feature of the end‐effector and is designed with a double‐bilayer structure. With this structure, the RMS can perform an opening and closing motion in different environments. Figure 1B demonstrates different modes that can be realized by the modular microrobots. Various applications can be done using different end‐effectors with an identical magnetic base. For example, pushing with only one sphere is obtained with a tipped end‐effector (Mode 1) while with an end‐effector having a larger opening the microrobot can push multiple spheres (Mode 2).

### 2.2 Simulations of the Responsive Mating Component

In order to understand the working mechanism of the RMCs, simulations based on finite element analysis (FEA) are performed for qualitative studies of the proposed modular microrobots. The simulations are achieved using commercial FEA software ABAQUS 2018. In these simulations, the non‐responsive parts are simulated as neo‐hookean materials with *C*_10_ = 0.015 MPa and *D*_1_ = 10 MPa^−1^ (properties used to define the neo‐hookean materials in ABAQUS considering compressibility) while the responsive parts are implemented using the user‐defined hyperelastic material subroutine UHYPER for environmental responsiveness. The final form of the free energy function used for the UHYPER implementation is given as:

(1) $$\begin{array}{cc} W & =\frac{1}{2}Nv\left[\lambda_{0}^{-1}{J^{\prime }}^{\frac{2}{3}}\bar{I_{1}}-3\lambda_{0}^{-3}-2\lambda_{0}^{-1}\ln\left(\lambda_{0}^{3}J^{\prime }\right)\right]\\ & +\left(J^{\prime }-\lambda_{0}^{-3}\right)\ln\left(\frac{J^{\prime }}{J^{\prime }-\lambda_{0}^{-3}}\right)-\frac{\chi }{\lambda_{0}^{6}J^{\prime }}-\frac{\mu }{kT}\left(J^{\prime }-\lambda_{0}^{-3}\right) \end{array}$$

after introducing a Legendre transform and using the free‐swelling state as the reference state^[^ ^22^ ^]^ where *J* and *J*′ are the volume ratios to the dry and free‐swelling states, respectively, and $J=\det F=\lambda_{0}^{3}J^{\prime }$ and $\bar{I_{1}}$ is the invariant of the deformation gradient **F**. *Nv* is a dimensionless parameter representing the number of polymer chains at the dry state, χ is the interaction parameter characterizing the interactions between the polymer matrix and the solvent, and µ is the chemical potential between the polymer matrix and the solvent before the system reaches equilibrium. The material properties used for the simulation are used with *Nv* = 0.0854, λ_0_ = 2.2617, and χ = ‐0.7363, respectively.^[^ ^34^ ^]^ Discussions of the simulation results can be found in the following sections accompanied by the corresponding designs of different mating components.

### 2.3 Fabrication and Validation of the Responsive Mating Components

The RMCs are fabricated using a commercial TPP system (Photonic Professional GT2, Nanoscribe GmbH) using a 63× objective with a poly‐(*N*‐isopropylacrylamide) (PNIPAM) hydrogel material. The preparation of the hydrogel precursor can be found in the Experimental Section. During the hydrogel printing, the oil‐immersion mode is adopted with a droplet of the precursor applied on a circular coverslip with a diameter of 30 mm. Immersion oil is applied between the coverslip and the objective. The swelling and deswelling ratios of the hydrogels are defined as the ratio of the resulting stimulated size to the designed size. If the resulting stimulated size is larger than the designed size, a swelling ratio is used, and vice versa for the deswelling ratio.

**Figure 2:**
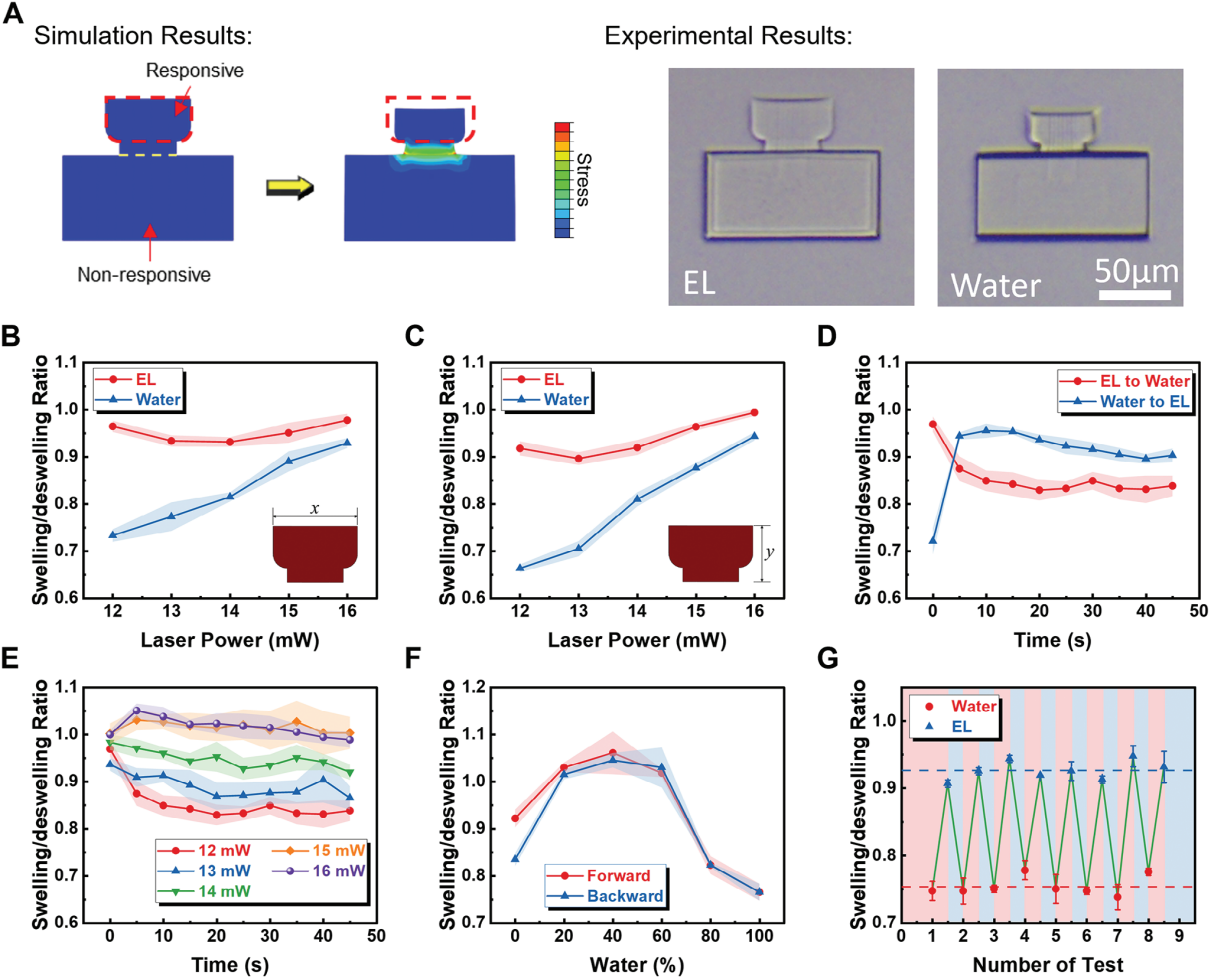
Swelling behaviors of the responsive mating designs with the Type 1 design. A) Qualitative simulation of deswelling of the mating component. B) Swelling ratio of the responsive mating designs with different printing LP along the *x*‐direction at equilibrium states with five samples (*n* = 5). (C) Swelling ratio of the responsive mating designs with different printing LP along the *y*‐direction at equilibrium states with *n* = 5. The data in (B) and (C) are collected right after the structures are printed and well‐developed for minimal residue. D) Transition swelling of the responsive mating design (LP = 13 mW) from EL to water and the corresponding reverse behaviors with *n* = 5. E) Transition swelling of the responsive mating designs with different LPs from EL to water with *n* = 5. F) Deswelling of the printed responsive mating design with an LP = 12 mW in different chemical environments (different compositions of water and EL) with *n* = 5. G) Repeatability of shape (deswelling ratio) change of the mating component between 100% water and EL (0% water) with *n* = 5. Error bars in (B–F) are represented with shaded areas with standard deviations (SD).

Using the finite element simulation, a qualitative validation is first performed to prove the feasibility of the RMC for the Type 1 microrobot as shown in **Figure** **2**A. As can be seen from the result, the inner stress within the RMC (red dash region) is at a low level maintaining minimal energy. However, the connecting region between the responsive and non‐responsive materials presents a high stress that may cause delamination if the two materials are not compatible. Figure 2A also provides experimental results of a printed sample deforming in different environments showing good agreement with the simulation. To gain a deeper understanding of the RMC, various samples are printed and tested with different laser powers (LPs), which result in different cross‐linking densities, printed with a non‐magnetic base. All RMCs are printed with a laser scanning speed (SS) of 8 mms^‐1^. The LP and SS together contribute to the overall dose polymerizing the hydrogel. As can be seen in Figure 2B,C, the designs printed with different LPs maintained close to the designed sizes (60 and 40 µm for *x* and *y* directions, respectively) with swelling ratios all above 90% in EL. However, there is a slight drop as the LP is decreased. The swelling ratio has a significant drop along both the *x* and *y* directions in water when the LP becomes smaller. Even though the deswelling ratio in *y* is slightly lower than in *x*, the size change in *x* is more important for the mating process. These large deformations provide the feasibility for mating different modules without a bilayer structure to enhance shape change as used in the Type 2 design. For structures printed with an LP less than 12 mW, they are either too soft to maintain good structural stability or the dose is below the cross‐linking threshold for the same SS. Therefore, 12 mW is adopted for the responsive part. For the non‐responsive part, if we keep increasing the LP, one can get highly cross‐linked structures having a swelling ratio of ≈1 in water as shown in the experimental results of Figure 2A. However, since the non‐responsive will be replaced by a much stiffer SU‐8 material when fabricating the magnetic base, the non‐responsive hydrogel is not further tested in this section.

The transition swelling behaviors of a sample printed with an LP of 12 mW between EL and water are also studied, for both the forward and backward swelling, as shown in Figure 2D. The swelling/deswelling from water to EL is fast within 5s to a relatively stable state. However, the transition from EL to water is much slower. The deswelling ratio is expected to be at around the equilibrium state while it only reaches 0.838 after 45 s. The speed of deswelling decreased as the chemical potential µ decreased. Stable data are measured after an hour. However, the transition measurement could be delayed due to the adhesion between the sample and the substrate. Figure 2E shows that the transition speed of deswelling is decreased with LPs. Figure 2F shows a sample printed with 12 mW of LP in different solvents (with different compositions of water and EL) at the equilibrium state. For convenience, different compositions of water and EL are referred to as percentage water, e.g., 40% water is a mixture of 40% of water and 60% of EL. As depicted in the figure, a peak is found at around 40% of water showing a swelling effect with a ratio higher than 1. The repeatability of the swelling/deswelling of the responsive part is tested for eight cycles as shown in Figure 2G. For eight cycles, the average deswelling ratio is 0.927 in pure EL and 0.753 in water. The results show a good consistency for repeatable use.

**Figure 3:**
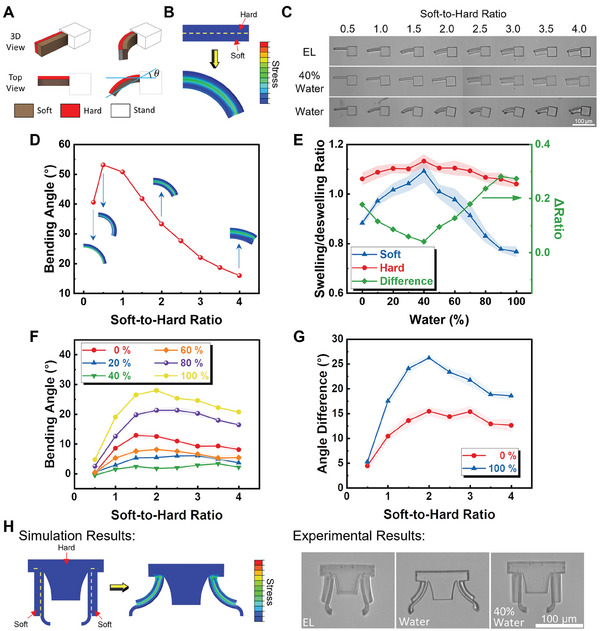
Bending behavior of bilayer structures with different thickness ratios. A) Systematic of the suspended bilayer structures. B) Qualitative simulation of a bilayer structure. C) Images of bilayer structures in environments with different EL/water compositions. D) Bending angles measured based on the simulation results with *n* = 5. E) Swelling ratios of the hard and soft layers (left axis), respectively, and the difference of swelling ratios between the two layers (right axis). F) Bending angle of the bilayer structures under different EL/water compositions with *n* = 5. G) Difference of bending angles of the bilayer structures in different environments with *n* = 5. The data are the differences between the labeled environments with respect to 40% water. H) Qualitative simulation and experimental results of the doubled‐bilayer design for end‐effectors for Type 2 microrobots. Error bars in (E–G) are represented with shaded areas with SD of five samples.

The Type 2 microrobot has a double‐bilayer structure to achieve open and closed configurations. Before applying the printing parameters to print the end‐effectors, some general tests with a single bilayer structure have been done to assist the designs. The bending behavior of the bilayer structures is tested with different thickness ratios of responsive (soft) to non‐responsive (hard) layers. The 3D design of the suspended bilayer structure is provided in **Figure** **3**A with a hard layer with dimensions of 59 µm × 6 µm × 20 µm. The bilayer structures are extruded and suspended, to minimize adhesion for bending, from a fixed base. The bending angle is defined as θ. A qualitative simulation, presented in Figure 3B, for this bilayer structure is also performed to verify the experimental phenomenon. Figure 3C shows the bending results of bilayer structures printed with different soft‐to‐hard thickness ratios in different water/EL compositions. The soft layers are printed with an LP of 12 mW and an SS of 8 mms^‐1^ while the hard layers are printed with an LP of 27.5 mW and a SS of 10 mms^‐1^. It can be seen that the bending angles of the suspended bilayers vary for different thickness ratios and chemical environments. The bending angles with different thickness ratios are also studied with simulation and are presented in Figure 3D showing a peak bending angle for a soft‐to‐hard thickness ratio of 0.5. With a smaller thickness ratio than this, the soft layer is too thin to generate enough stiffness mismatch to obtain a significant bending. Analytical analysis using the modified Timoshenko bimorph beam theory also proves the existence of the peak.^[^ ^14^ ^]^ The radii can be calculated as

(2) $$R=\frac{\left(h_{1}+h_{2}\right)\left(8{\left(1+m\right)}^{2}+\left(1+mn\right)\left(m^{2}+\frac{1}{mn}\right)\right)}{6\varepsilon {\left(1+m\right)}^{2}}$$

where *n* is the ratio of elastic modulus of the hard and soft layers, *m* is the thickness of the hard (*h*_1_ = 6 µm) and soft (*h*_2_) layers, and ɛ is the difference of expansion coefficient of those two layers. Using *n* = 2 and ɛ = 0.3, based on previously reported material properties of similar hydrogels with different cross‐linking density^[^ ^34^ ^]^ and the difference in deswelling ratios displayed in Figure 3E, will give a local minimum radius at a soft‐to‐hard ratio corresponding to 0.25. For a certain length of the bilayer structure, the smaller the radius the larger the bending angle, which is the peak. Experimental measurements are provided in Figure 3F. As can be seen, the bending angles decreased as the water content of the environment increased up to 40%. When the water content reaches 40%, the soft responsive structure is restored to its designed shape. Then, as you keep increasing the water content to 100%, a more significant bending angle emerges, as expected. Figure 3E provides the deswelling ratios of the soft and hard hydrogels in different compositions of solvents along with the difference between them. As can be seen from the figure, even though both the soft and hard hydrogels present a larger size than the designed size, the difference in the swelling ratio is minimized to 0.04 and therefore the bending angle of the bilayer structure is close to 0. Both directions deviating from 40% water will give a significant difference in the swelling ratios thus resulting in a bending deformation. The maximum bending appears when the environment is close to pure water. The largest bending angle appears between the thickness ratio of 1.5 to 2.5 for all compositions. The appearance of the peak is in agreement with the simulation result. Figure 3G gives the angle differences between 100% water and 40% water and between 0% water and 40% water, where 40% water is a reference state that gives a nearly zero bending angle. Using the compositions providing the largest (pure water) and smallest (40% water) bending angles, the angle difference can be as high as 27° with a thickness ratio of ≈2.

After getting the bending result from the suspending bilayer structures, the obtained optimal thickness ratio of 2 is adopted for the end‐effector design. Figure 3H shows the printed preliminary design of an end‐effector with two bilayer structures working simultaneously like a gripper, verified by a qualitative simulation as well. This gripper design is used as a locking mechanism with the magnetic base in the Type 2 design. The end‐effector stays in its designed shape when it is in an environment with 40% of water so that it can successfully lock the base. When the end‐effector is in pure water, the bilayer structures bend and provide a significant opening to disengage the base.

### 2.4 Fabrication of the Modular Microrobots

**Figure 4:**
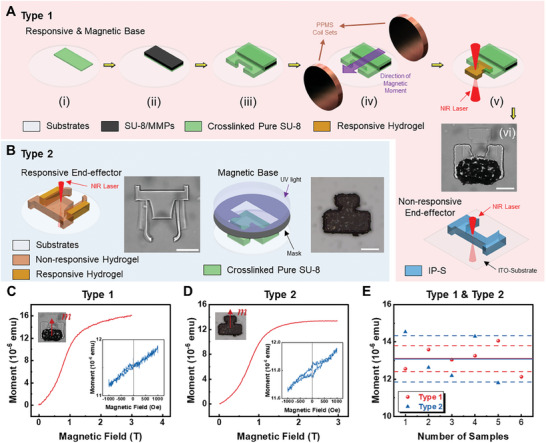
Fabrication of different types of modular microrobots. A) Fabrication process of the magnetic base for Type 1 microrobots containing an RMC and a non‐responsive end‐effector. B) Fabrication processes of the Type 2 design with a responsive end‐effector and magnetic base. C) Magnetization of the magnetic base of a Type 1 microrobot. D) Magnetization of the magnetic base of a Type 2 microrobot. Red curves in (C) and (D) are moments measured during the magnetizations, while the blue curves in the insets are remanence moments measured with four quadrants with a field of 1000 Oe. E) Remanent moments of multiple samples of the Type 1 and Type 2 magnetic bases. The solid lines represent the average values and the dash lines show the band around the averages with one σ (SD). All scale bars: 50 µm.

The fabrication processes of the modular microrobots are shown in **Figure** **4**, which combine the traditional photolithography and the TPP technique. The Type 1 responsive and magnetic base (RMB) is fabricated via the route depicted in Figure 4A. First, (i) a rectangular pure SU‐8 layer is patterned on a circular coverslip with a diameter of 30 mm for better adhesion for the next layer. Then, (ii) a rectangular magnetic piece is photolithographed with a SU‐8/magnetic microparticles (MMPs) mixture followed by (iii) another larger layer of pure SU‐8 covering the magnetic piece with an additional protruding part for hydrogel printing. The SU‐8/MMPs mixture is prepared with a 1 to 1 weight ratio of both pure SU‐8 and MMPs followed by a high‐speed mixing at 7000 rpm. The second layer of pure SU‐8 is designed with an opening for hydrogel printing inside the base to avoid out‐of‐plane bending along the connection line between the soft and hard materials. Before printing the hydrogel RMC, the base is transferred to a physical properties measurement system (PPMS, Quantum Design Dynacool) to be magnetized under a 3 T field (iv); otherwise, the base shows no significant magnetic moment to be controlled under a magnetic field. This is because magnetic microparticles have no or little apparent magnetic moment before the magnetization since the particles are randomly mixed. The application of a 3 T magnetic field is able to pull the magnetic moment of each microparticle to an identical direction resulting in a significant moment. The PNIPAM hydrogel is then printed using the TPP system with a 63× oil‐immersion objective with an LP of 12 mW and an SS of 8 mms^‐1^ (v) using the oil‐immersion mode. After the printing process, the Type1 RMB is developed with isopropyl alcohol (IPA) for an hour to remove any residue. An example of the RMB is shown in Figure 4A (vi). The non‐responsive end‐effectors for the responsive base are simply printed by the TPP process using the 25× objective with the dip‐in mode using the two‐photon resist IP‐S on an indium‐tin‐oxide (ITO)‐coated substrate. Both the resist and the substrate are obtained from the same company as the TPP system.

The fabrication of the Type 2 inverse design is simpler with the responsive end‐effector achieved by the TPP process while the magnetic base is obtained by photolithography, as shown in Figure 4B. The responsive end‐effector has two bilayer regions resulting in an opening and closing motion. The printing parameters of the bilayers are the same as shown in Figure 3 with an LP of 12 mW and an SS of 8 mms^‐1^ for soft regions and an LP of 27.5 mW and an SS of 10 mms^‐1^ for the hard regions of the end‐effector. The magnetic base is achieved by photolithography of three layers of SU‐8, SU‐8/MMPs, and SU‐8, respectively, using the same mask design. The magnetic base is also magnetized by the PPMS with a magnetic field of 3 T. Both the base and end‐effector for the two types of modular microrobots are designed with a width of 120 µm for an overall footprint size of 120 µm × 200 µm after mating. All features of the microrobots are printed with a thickness of 20 µm and the parts that are obtained by photolithography have a similar thickness.

Figure 4C, D gives the magnetization data of both the bases for Type 1 and Type 2. As can be seen from the red curves, a 3 T magnetic field is enough to achieve saturation. The remanent moments for both bases are tested using a much smaller field (1000 Oe for four quadrants) after the magnetization and the results are presented as insets in Figure 4C,D. As expected, the magnetic base for the Type 1 design has a similar magnetic moment (1.310× 10^−5^ emu) to the Type 2 (1.308× 10^−5^ emu) as the areas of the regions containing magnetic materials are similar. Multiple samples were tested for both Type 1 and Type 2 bases and variations in the magnetic moment values were less than 15%, as shown in Figure 4E.

### 2.5 Functional Performance Validation

**Figure 5:**
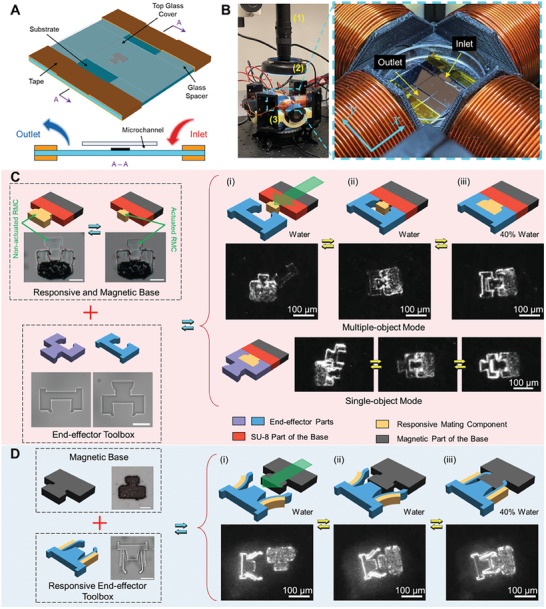
Schematic of the modular microrobot with switchable end‐effectors. A) Design of the microchannel used for testing the modular microrobots. B) Actuation system for generating gradient magnetic field and the prepared microchannel placed in the electromagnetic coils. The microscope, light source, and coils are labeled from 1 to 3, respectively. C) Mating processes of the Type 1 microrobot with end‐effectors for single‐pushing and multiple‐pushing. D) Mating process of the Type 2 microrobot. All other scale bars: 50 µm.

The schematic of the concept of operations and experimental setup for the modular microrobots are presented in **Figure** **5**. The validation experiments are performed in a simple microfluidic environment with a substrate and a coverslip that are spaced by another two coverslips at either end as shown in Figure 5A. The glass spacers are fixed using tape. Two coverslip spacing layers, each with an ideal thickness of 150 µm produce a microchannel with a height of 300 µm. The solvents are exchanged using a pipette by the capillary effect from one side (inlet) to the other side (outlet). The magnetic bases are controlled using a custom‐built electromagnetic coil system capable of generating gradient fields with a setup shown in Figure 5B. Two pairs of coils are able to generate magnetic gradients along the *x* and *y* directions. The prepared microchannel placed in the coil system is also shown in Figure 5B with the inlet and outlet labeled. The locomotion of the base is controlled with a 3D joystick that is able to produce *x*, *y*, and rotational motions.

The demonstrations of the use of the Type 1 microrobot are shown in Figure 5C. Two different types of end‐effectors are available for either multiple‐object pushing or single‐object pushing. Meanwhile, the fabricated RMB is tested for the non‐actuated RMC and actuated RMC states using 40% water and pure water. A significant shrinkage is found as expected. The mating process can be separated into three steps. The first step (i) is where the RMB is disengaged and located away from the end‐effector in water. Then the second step (ii) is the mating process where the RMB is controlled in pure water into the mating position. The final step (iii) is when the RMC returns to the non‐actuated state and the end‐effector and base are locked together as the environment changes from water to 40% water. The process is the same for both the single‐object and multiple‐object pushing end‐effectors. The illustration of the concept of operations of the Type 2 microrobot is given in Figure 5D. However, only one type of responsive end‐effector is demonstrated. The focus of these tests was on verifying the Type 2 mating mechanism. The overall mating process is similar to Type 1. The difference is in the locking mechanism between the end‐effector and the base, achieved via clamping. The responsive end‐effector stays open in water during the period when the base is away (i) and moved to the mating‐ready position (ii). The mating process is completed by changing the solvent to 40% water to trigger the closing motion (iii).

### 2.6 Micromanipulation

**Figure 6:**
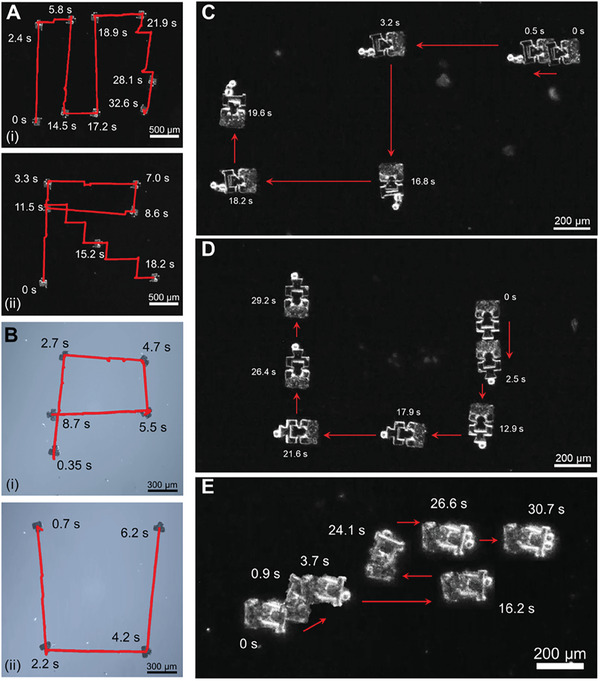
Locomotion tests and micromanipulation tasks for different types of modular microrobots. A) Controlled trajectories of the letters M and R for MicroRobot with a Type 1 magnetic base under the environment of 40% water. B) Controlled trajectories of the letters P and U for Purdue University with a Type 2 magnetic base in water. C) Manipulation of a sphere cluster using the Type 1 microrobot with an end‐effector for multiple‐object pushing. D) Manipulation of a sphere using the Type 1 microrobot with an end‐effector for single‐object pushing. E) Manipulation of multiple spheres at different time frames using the Type 2 microrobot with an end‐effector for multiple‐object pushing.

After successfully mating their magnetic bases to a particular end‐effector, the modular microrobots can be used for micromanipulation tasks. First, controlled propulsion along prescribed trajectories of the magnetic bases is investigated manually. **Figure** **6**A shows the controlled experimental trajectories achieving letters of M (i) and R (ii), which present the abbreviation of MicroRobot, using a Type 1 base in 40% water. The Type 1 base has a RMC made from hydrogels, while the Type 2 base does not. As a result, it is not easy for the Type 1 base to move in 100% water. A slow speed or a choppy motion is observed because of strong adhesion from the RMC and surface tension in water. However, since a pure water environment is only needed during the mating process and the manipulation tasks are achieved in 40% of water, the controlled locomotion tests are performed in an environment of 40% water. The mating process in water can be realized using rotational motions because rotational motions are more efficient. Due to the limitation of the control system, off‐axis or diagonal motions are achieved by shorter motions decomposed into *x* and *y* directions, as can be seen in Figure 6A ii. Any manufacturing defects or adhesive spots with the substrate will give rise to disturbances in the trajectory. The Type 2 base is able to move in 100% water since the base has a stronger magnetic moment and does have a hydrogel component that could introduce possible substrate adhesion. Comparing the time frames in Figure 6A,B, the Type 1 base shows a faster speed to reach targeted locations even in water. The letters P and U represent Purdue University are drawn with the trajectories in Figure 6 Bi,ii, respectively. However, even though both types of microrobots can move on silicon or glass substrates, a silicon substrate is used under the glass substrate since the end‐effectors for both types and the RMC of the Type 1 base are transparent. Therefore, the dark silicon background under a microscope provides better imaging results with improved contrast.

Micromanipulation tasks achieved by Type 1 and Type 2 modular microrobots are demonstrated in Figure 6C–E. Figure 6C showcases the Type 1 microrobot with a multiple‐object pushing end‐effector pushing a sphere cluster. Figure 6D presents the Type 1 microrobot manipulating a single 30 µm diameter sphere using the single‐object pushing end‐effector. The pushing of the sphere cluster proves that the Type 1 RMB is strong enough to move both the attached end‐effector and multiple spheres. Figure 6E demonstrates the use of a Type 2 microrobot the multiple‐object pushing end‐effector to push multiple spheres sequentially. The Type 2 microrobot successfully picks up sphere 1 at the timeframe *t* = 3.7 s and transports it until *t* = 24.1 s. It then picks up sphere 2 at *t* = 26.6 s and transports it until *t* = 30.7 s. After manipulating the spheres to the target position, they can be released by applying a simultaneous backward and rotational motion. Using this technique, the objects are typically released roughly within one body‐length of the robot from the target position.

### 2.7 End‐Effector Detachment

**Figure 7:**
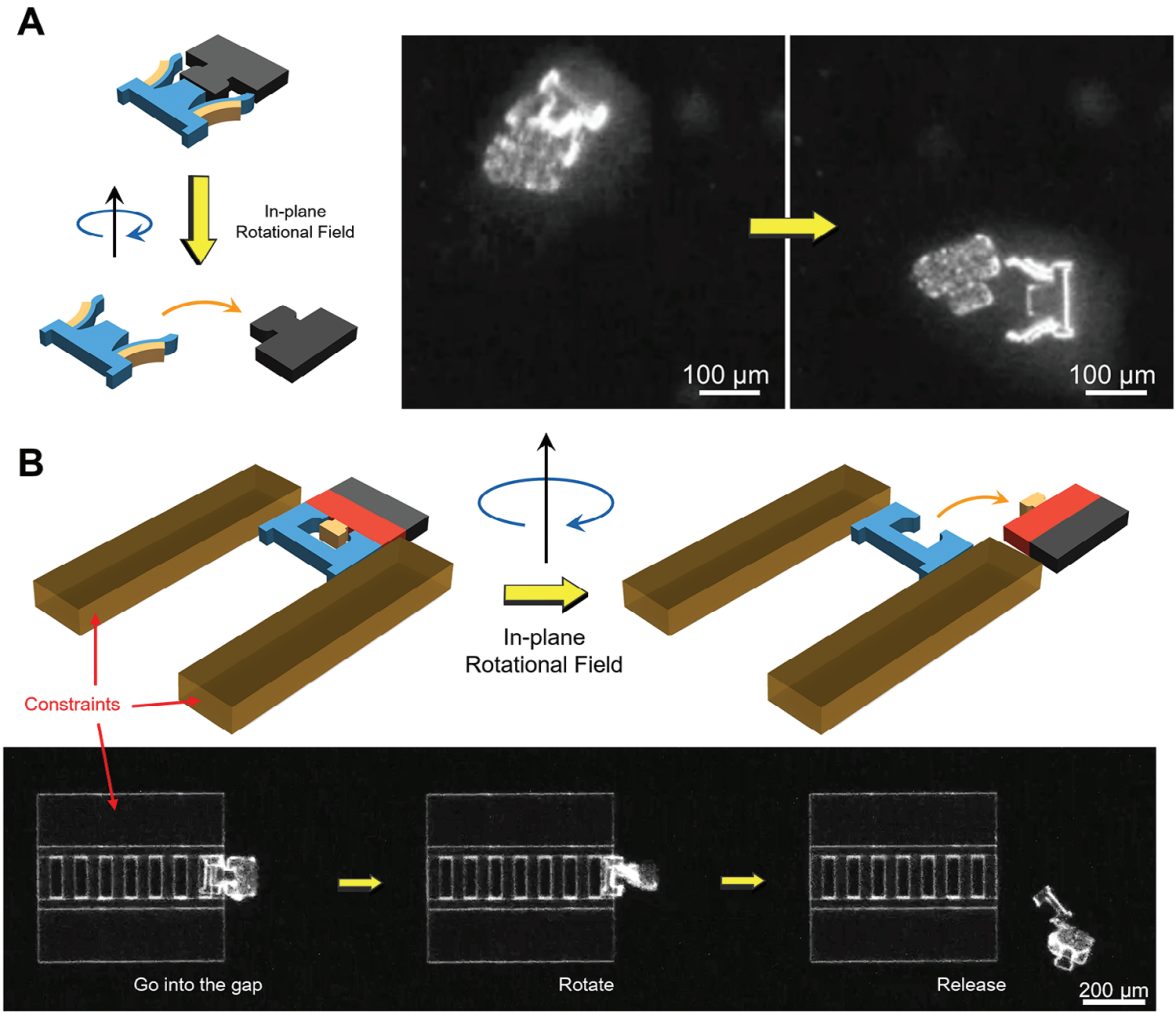
Detachment of end‐effectors of the modular microrobots. A) Detachment of the Type 2 modular microrobot using a rotational field in water. B) Detachment of the Type 1 modular microrobot using a rotational field with the assistance of a channeled constraint in water.

We have demonstrated the mating process and micromanipulation with the two different types of modular microrobots. However, it is essential to prove that the magnetic base can be detached from the end‐effector for end‐effector exchange. **Figure** **7** illustrates the detachment process for both types of microrobots. The Type 2 microrobot is presented first in Figure 7A. The base can be detached easily from the end‐effector as long as the double‐bilayer structure is open in water by using a rotational motion. This is because when stimulated, the double‐bilayer structure produces a wide opening around the male feature of the base. The situation for the Type 1 microrobot is different. Even the male RMC becomes smaller due to shrinkage once stimulated, the RMC is still surrounded by the female feature of the end‐effector with a small tolerance gap. This results in a strong surface tension between the components, especially in 100% water. Therefore, this surface tension and inherent adhesion between the end‐effector and the base prevent the detachment using a simple rotational motion. Therefore, assistance is required for the detachment of the Type 1 microrobot. As can be seen in Figure 7B, two large rectangular pieces are printed on a silicon substrate creating the microchannel. The gap between the two pieces is used to provide a constraint for the end‐effectors. A top enclosure is also introduced to prevent any possible out‐of‐plane motions during the detachment. A rotational motion is performed after moving the end‐effector inside the gap. Once the rotational motion of the end‐effector is constrained by the walls, the end‐effector and the base can be successfully detached via a rotational motion of the RMB.

## 3 Conclusion

In this paper, the design and fabrication of two types of modular microrobots are proposed by using both traditional photolithography and two‐photon polymerization 3D printing techniques. The magnetic base and the end‐effector of the modular microrobot are connected with a male‐female design, which is realized through the use of stimuli‐responsive hydrogels (responsive mating components). Two types of modular microrobots based on the location of responsive mating components are discussed. The mating process of the magnetic bases and the end‐effectors for both types are successfully validated in different solvents that trigger the process. Different micromanipulation tasks using the proposed modular microrobots are demonstrated with both types of microrobots. The detachment process of the magnetic bases from the end‐effectors is also demonstrated. We report the successful fabrication and demonstration of functions of the modular microrobot with an overall footprint of 120 µm × 200 µm that is able to perform different tasks with the same magnetic base by using different end‐effectors. In the meantime, the separation of the design and fabrication complexities is successfully demonstrated. A significant example of the advantage of using the proposed modular design is the capability of using micro‐force sensing end‐effectors^[^ ^9^ ^]^ with different measurement ranges avoiding using multiple microrobots that are hard to control individually under a global magnetic field.

## 4 Experimental Section

### Materials

Acrylic acid (AAc), polyvinylpyrrolidone (PVP), ethyl lactate (EL), triethanolamine (TEA), 4,4'‐bis(diethylamino)benzophenone (EMK), and *N*,*N*‐dimethylformamide (DMF) were purchased from Sigma–Aldrich. *N*‐Isopropylacrylamide (NIPAM) and dipentaerythritol pentaacrylate (DPEPA) were obtained from Scientific Polymer Products Inc. and Alfa Chemistry, respectively. The photoresist SU‐8 2025 was purchased from Fischer Scientific. The neodymium magnetic microparticles (MMPs, MQFP‐B) with an average diameter of 5 µm were obtained from Magnequench. The IP‐S TPP resin was obtained from Nanoscribe GmbH.

### Preparation of Precursors

The hydrogel precursor for printing was prepared by mixing chemicals in two steps as described in Ref. [37]. In brief, 1.6 g of NIPAM, 0.8 mL of AAc, and 0.15 g PVP were dissolved in 1 mL of EL followed by vigorous stirring for complete dissolution. Then 2.5 mL of the solution obtained above was mixed with 0.4 mL of DPEPA, 0.5 mL of TEA, and 100 µL of EMK/DMF solution (1 to 4 weight ratio). Complete dissolution was achieved by mixing with a magnetic stirrer. The SU‐8/MMPs mixture was prepared with a 1 to 1 weight ratio of both pure SU‐8 and MMPs followed by a high‐speed mixing at 7000 rpm.

### Sample Preparation for Validation

Both of the Type 1 and Type 2 RMCs were first validated by printing the structures with the TPP system using the hydrogel precursor prepared above with a 63× oil‐immersion objective. For the validations, both types of RMCs were printed with soft and hard parts. The soft part was responsive to different solvents while the hard part was barely responsive and used as fixtures or as a supporting layer for bending deformations. The soft layers were printed with an LP of 12 mW and an SS of 8 mms^‐1^ while the hard layers are printed with an LP of 27.5 mW and a SS of 10 mms^‐1^.

### Fabrication of the Modular Microrobots

The Type 1 RMB was fabricated following the steps below. First, a rectangular pure SU‐8 layer was patterned on a circular coverslip with a diameter of 30 mm. Then, a rectangular magnetic piece was photolithographed with a SU‐8/MMPs mixture followed by another larger layer of pure SU‐8 covering the magnetic piece with an additional protruding part for hydrogel printing. The second layer of pure SU‐8 was designed with an opening for hydrogel printing inside the base to avoid out‐of‐plane bending along the connection line between the soft and hard materials. Before printing the hydrogel RMC, the base was transferred to a PPMS to be magnetized under a 3 T field. The PNIPAM hydrogel was then printed using the TPP system with a 63× oil‐immersion objective with an LP of 12 mW and an SS of 8 mms^‐1^ using the oil‐immersion mode. After the printing process, the Type1 RMB was developed with IPA for an hour to remove any residue. The non‐responsive end‐effectors for the responsive base were simply printed by the TPP process using the 25× objective with the dip‐in mode using the IP‐S resist on an ITO‐coated substrate. Both the resist and the substrate were obtained from the same company as the TPP system.

The fabrication of Type 2 was given as follows. The responsive end‐effector had two bilayer structures that were printed with an LP of 12 mW and an SS of 8 mms^‐1^ for soft regions and an LP of 27.5 mW and an SS of 10 mms^‐1^ for the hard regions. The magnetic base was achieved by photolithography of three layers of SU‐8, SU‐8/MMPs, and SU‐8, respectively, using the same mask design. Then, the magnetic base was also magnetized by the PPMS with a magnetic field of 3 T. All the obtained end‐effectors and the magnetic bases were released and transferred to the testing chamber using a fine tweezer.

### Experimental Platform

Both the validation and manipulation experiments were performed in a simple microfluidic environment with a substrate and a coverslip that were spaced by another two coverslips at either end. The glass spacers were fixed using tape. Two coverslip spacing layers, each with an ideal thickness of 150 µm produce a microchannel with a height of 300 µm. The solvents were exchanged using a pipette by the capillary effect from one side (inlet) to the other side (outlet). The magnetic bases were controlled using a custom‐built electromagnetic coil system capable of generating gradient fields with two pairs of coils that could generate magnetic gradients along the *x* and *y* directions. The prepared microchannel was placed at the center of the coil system. The locomotion of the base was controlled with a 3D joystick that could produce *x*, *y*, and rotational motions by controlling the field generated by the coil system.

### Testing accessories

The spheres and the constraints used for micromanipulation tasks and Type 2 detachments, respectively, were printed with the IP‐S resin using the Nanoscribe TPP system with a 25× objective.

## Conflict of Interest

The authors declare no conflict of interest.

## Supporting information


Supplemental Video 1

Supplemental Video 2

Supplemental Video 3

Supplemental Video 4

## Data Availability

The data that support the findings of this study are available from the corresponding author upon reasonable request.
